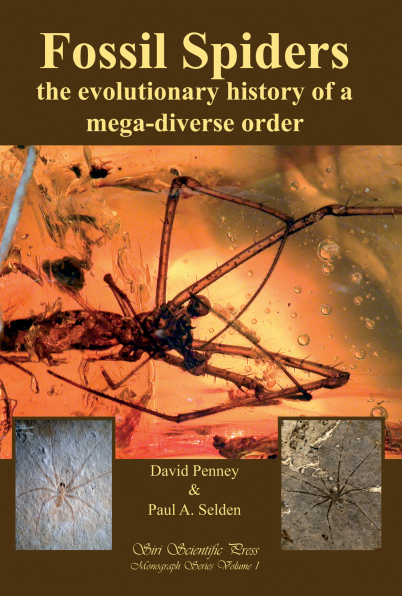# Book review: Penney D, Selden PA (2011) Fossil Spiders: the evolutionary history of a mega-diverse order. Monograph Series, Volume 1. Siri Scientific Press, Manchester, 128 pp., 87 photographs (hardback)

**DOI:** 10.3897/zookeys.119.1736

**Published:** 2011-07-15

**Authors:** Yuri M. Marusik

**Affiliations:** Institute for Biological Problems of the North, Portovaya Str. 18, Magadan 685000 Russia

Prior to the publication of the book reviewed here, the only books dealing specifically with fossil spiders have been primarily taxonomic monographs, such as those on Dominican and Baltic amber inclusions by (Wunderlich (1988, 2004), or broader palaeobiological studies on a particular assemblage, such as Dominican amber spiders by Penney (2008). The coverage of fossil spiders in books on extant spiders is usually restricted to one or two pages at most, and more often than not these include serious errors. This new book is quite different and fills a long standing void in the arachnological literature.

The book begins with an introduction to spiders from a palaeontological perspective, putting their geological longevity into context of the other known fossil and extant arachnid orders, including comparative data for numbers of described extant and fossil species for all orders. The relative diversity of spider sub-orders today is compared with that of the past. The authors provide reasoned support for the importance of fossil spiders in addressing large-scale palaeobiological questions on a global scale, and over long periods of time, including how information on past changes in biogeographical distributions can be correlated with changes in palaeoclimatological factors and the potential of this for predicting the consequences of current climate change. Information is also provided on how the fossil spiders are dated.

The fossil record of spiders is discussed with regard to non-amber fossils (there is a table of 51 important localities with references), including the different modes of preservation with photographic examples (often of holotypes) throughout. The amber spider fossil record follows a similar format with a table of 42 important localities. There is also a section on fossilized spider silk. An important aspect of interpreting fossil assemblages is an understanding of bias in the fossil record and this is covered for both amber and non-amber deposits.

Next follows a highly informative chapter on techniques for the preparation and study of fossil spiders, which will be of great use for new students of palaeoarachnology. It includes techniques for preparing both amber and non-amber fossils, including how to obtain the best photographs, and also the application of new imaging techniques which allow non-destructive digital dissection of the fossils, such as x-ray computed and synchrotron technology. The images included here are quite exceptional. This is followed by a discussion of the problems associated with identifying and naming fossil spiders, particularly in a neontological context, including critical examples of techniques recently proposed to assist in this process.

There is a chapter on the strictly fossil spider families, which lists all known species and their deposit of origin.The taxonomic status of each family is discussed and it is noted that many are in need of revision. Again, this section includes good photographs including of holotypes.

Approximately half way through, the focus of the book switches from understanding and interpreting the fossil spiders to the application of the palaeontological data. Much use is made of the spider evolutionary tree, which was created by superimposing the cladogram of Araneae over the geological time scale and calibrating it with described fossils. This tree is up-to-date and even includes the recently erected family Penestomidae and the recent synonymy of Microphocommatidae with Anapidae. There is a handy table referencing the oldest fossil of each of the 73 extant spider families known in the fossil record, used to create the evolutionary tree. Topics in this section include originations and radiations (with particular emphasis on diverse families today such as Salticidae, Theridiidae and Lycosidae), mass extinctions, and predator-prey co-radiations, including updated analyses (based on new data) of previously published work by the authors.

The last chapter concerns how fossil spiders can contribute to our understanding of past and present biogeography. The authors present some rather remarkable examples of disjunct distributions between the fossil and extant spider faunas. A table of spider family distributions over time, including recent, Cenozoic and Mesozoic distributions presents the available data in a user-friendly fashion. Importantly, the authors note that it is just as important to look at which families do not appear in the fossil record and provide a table of data for these also, and discuss possible reasons for their exclusion. The book ends with a comprehensive reference section of some 265 entries and an index to family citations. There is a handy reference figure of the geological timescale on page 6.

In conclusion, this is an absolutely unique book in the spider literature to date. It is authoritatively written by two of the leading researchers in this field and provides broad coverage of their combined 50 years experience and expert knowledge. The book is hard back and presented on high quality glossy paper. It is richly illustrated thoughout by numerous high quality photographs of fossil spiders. In conclusion, this book deserves a place on the shelves of the libraries of all professional and amateur arachnologists alike, in addition to those of invertebrate palaeontologists.

The book can be ordered via the author: by emailing siri.press@live.co.uk. Price £32.00 plus post and packing. For further details, including ordering online, etc. visit http://www.siriscientificpress.co.uk

**Figure:**